# Wealth and depression: A scoping review

**DOI:** 10.1002/brb3.2486

**Published:** 2022-02-08

**Authors:** Catherine K. Ettman, Gaelen P. Adam, Melissa A. Clark, Ira B. Wilson, Patrick M. Vivier, Sandro Galea

**Affiliations:** ^1^ Office of the Dean Boston University School of Public Health Boston Massachusetts USA; ^2^ Department of Health Services, Policy and Practice Brown University School of Public Health Providence Rhode Island USA; ^3^ Hassenfeld Child Health Innovation Institute Providence Rhode Island USA

**Keywords:** depression, mental health, review, social and economic factors, wealth

## Abstract

**Introduction:**

The inverse relation between income and depression is well established. Less is understood about the relation between wealth and depression. We therefore conducted a scoping review to answer the question: What is known from the existing literature about the relation between wealth and depression?

**Methods:**

We searched for studies and articles in Medline (via PubMed), Embase, PsycINFO, PsycArticles, EconLit, and SocINDEX from inception through July 19, 2020. Ninety‐six articles were included in our review. Key article characteristics were year of publication, sample size, country, study design, definition of depression, definition of wealth, and association between wealth and depression. Thirty‐two longitudinal articles were included in a detailed charted review.

**Results:**

Depression was defined in a relatively standard manner across articles. In contrast, definitions and measurements of wealth varied greatly. The majority of studies in the full review (*n* = 56, 58%) and half of the studies in the longitudinal charted review (*n* = 16, 50%) reported an inverse relation between wealth and depression. The longitudinal charted review showed that (1) macro‐economic events influenced depression, (2) wealth status influenced depression across the lifecourse, (3) wealth protected against depression in the face of stressors such as job loss, (4) subjective or psychosocial factors such as perception of wealth, relative comparison, and social status modified the relation between wealth and depression, and (5) savings interventions were successful in reducing depression and varied by context.

**Conclusion:**

These findings suggest that wealth should be included in our consideration of the forces that shape mental health.

## 1 INTRODUCTION

### 1.1 Background

Poor mental health is a leading cause of morbidity worldwide (Cohen & Galea, 2012; Malhi & Mann, 2018). Poor mental health is costly (Kessler, 2012), can lead to worse physical health (Richmond‐Rakerd et al., 2021), and affects both individuals experiencing poor mental health and their communities (Greenberg & Birnbaum, 2005). Multiple factors contribute to individual risk of poor mental health, ranging from genetic factors to individual behavior to features of an individual's context. Depression, in particular, is sensitive to the economic and social contexts that shape individual lives (Allen et al., 2014). Having financial resources can protect people from the stressors that drive depression and can lighten the blow of disruptions to daily living (Hammarström & Virtanen, 2019). Although there is abundant evidence that higher income is associated with better mental health (Kawachi et al., 2010; Patel et al., 2018), the relation between wealth and mental health is far less clear. By wealth, we refer to the total non‐income accumulated assets that persons own or have access to, including financial assets such as money in savings accounts or stocks and physical assets such as home ownership. Wealth can be passed intergenerationally and may be a better indicator of financial stability than income and may also better document inequities than income.

### 1.2 Rationale

There is little question that low income is one of the central determinants of health (Kawachi et al., 2010; Krieger, 1994; Krieger et al., 1997; O'Donnell et al., 2013). Relatedly, but not directly addressing the question of interest here, Patel and colleagues conducted a systematic review of the literature on income inequality and depression and found that increases in income inequality were associated with increases in depression. The effects varied across subpopulations, with women and low‐income persons experiencing worse impacts of income inequality on depression (Patel et al., 2018). However, a growing literature suggests that wealth may be an even more important factor in shaping health (Erixson, 2017). As a representation of accumulated assets over time, potentially shared intergenerationally, wealth reflects a feature of financial stability and socioeconomic advantage that can protect individuals throughout their lifecourse. While there have been several reviews aimed at better understanding what is known about the relation between wealth and physical or mental health (Baum, 2005; Braveman & Gottlieb, 2014; Pollack et al., 2007), we are not aware of any review that has specifically focused on the relation between wealth and depression. In a review of studies about wealth and a range of health outcomes, Pollack and colleagues identified six studies between 1990 and 2006 that explored socioeconomic indicators and depression (Pollack et al., 2007). However, the review did not focus specifically on depression and provided only high‐level summaries.

Two population level trends in particular motivate this review: an increase in wealth inequality due to the unequal economic burden created by the coronavirus disease 2019 (COVID‐19) pandemic and an increase in reporting of depressive symptoms during the COVID‐19 pandemic (Ettman et al., 2020a; Luo et al., 2020). Recent studies suggest that persons with low wealth may be at greater risk of depression when the COVID‐19 pandemic ends (Ettman, Cohen, Abdalla, et al., 2020; Ettman, Cohen, & Galea 2020; Ettman et al., 2020b; Ettman, Cohen, Vivier, et al., 2020). There remains, however, a lack of clarity about the potential effect of wealth on depression. Understanding how wealth relates to depression will be an important next step, and a useful tool for researchers and policy makers, as countries grapple with a high prevalence of depression and economic distress in the coming years.

### 1.3 Objectives

We conducted a scoping review to systematically understand the research about wealth and depression and to identify gaps in the literature about wealth and mental health. In particular, the goal of this study was to better understand the relation between depression and wealth as a liquid asset, as opposed to wealth in the form of home ownership or debt, which have been covered in other reviews (Richardson et al., 2013; Tsai, 2015; Tsai & Huang, 2019). Our research question was the following: what is known from the existing literature about the relation between wealth and depression?

## 2 METHODS

### 2.1 Protocol

We evaluated the peer‐reviewed published literature from the beginning of search functions through July 19, 2020 on financial assets and depression. We followed the Preferred Reporting Items for Systematic reviews and Meta‐Analyses extension for Scoping Reviews (PRISMA‐ScR) (Tricco et al., 2018) guidelines.

### 2.2 Eligibility criteria

Eligible studies were English‐language, quantitative analyses on human subjects that focused on objective financial indicators (e.g., wealth, assets, savings) and depression. We excluded articles solely about income, nonliquid assets (e.g., home ownership), and debt. The relation between home ownership and mental health has been well documented in other reviews (Tsai, 2015; Tsai & Huang, 2019; Vásquez‐Vera et al., 2017). Similarly, the relation between debt and mental health is complex and has been addressed elsewhere (Richardson et al., 2013). Studies were excluded if they did not feature depression or psychological distress. Studies were also excluded if they featured postpartum depression, since postpartum depression is a distinct clinical event that is separate in nature from other forms of depression (Di Florio & Meltzer‐Brody, 2015). The literature suggests differentiating between postpartum depression and depression experienced during other times of life, given their different pathways and unique features (Batt et al., 2020). We did not include studies with ecologic data for depression (i.e., we excluded studies that did not assess depression at the individual level).

### 2.3 Information sources

We searched the following databases from inception through July 19, 2020: Medline via Pubmed, Embase, PsycINFO, PsycArticles, EconLit, and SocINDEX. We exported the final search results to Zotero and EndNote, where duplicates were removed. Abstract screening was conducted using Abstrackr (Rathbone et al., 2015). The search strategy was created in partnership with a Medical Sciences Librarian; the full search strategy can be found in Appendix SA. The database search was supplemented by additional searches using Google Scholar and the snowball technique of reviewing references of seminal papers.

### 2.4 Selections of sources of evidence

**FIGURE 1:**
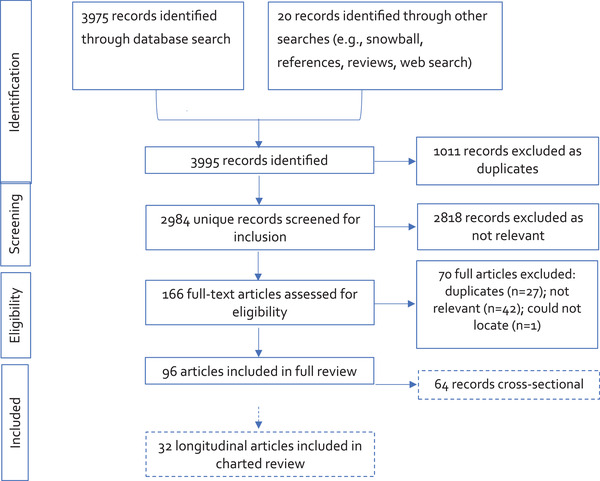
Preferred Reporting Items for Systematic Reviews and Meta‐Analyses (PRISMA) flowchart of study selection process

We reviewed all abstracts for relevance. CKE sequentially reviewed titles, abstracts, and full articles for inclusion in the final full review. We excluded duplicate articles and articles that did not meet eligibility criteria. Reasons for exclusion from the full review were the following: studies did not report on the relation between depression and wealth (*n* = 31), duplicates (*n* = 27), articles were not quantitative studies (*n* = 8), studies were not conducted on human subjects (*n* = 2), postpartum depression was measured (*n* = 1), and could not locate the full article (*n* = 1). A flowchart of the study selection process can be seen in Figure 1.

### 2.5 Data charting process

A chart for mapping the data was created to determine variables to extract. The data were charted and revised in an iterative fashion. The “charted review” refers to the subset of articles selected for detailed charting, in this case 32 longitudinal studies selected.

### 2.6 Data items

We organized the full review data (*n* = 96) by the year the article was written, sample size, country where the study was conducted, study design, definition of depression, definition of wealth, and key findings. We focused on a subset of the full review to include in the featured charted review, selecting the longitudinal studies (*n* = 32). In the longitudinal charted review, we mapped the year of publication, research question, study population, study design, definition of depression, depression of wealth, direction of association between wealth and depression, and conclusions. While cross‐sectional studies provide information about the association between wealth and depression, longitudinal studies with at least two time points can provide potential causal inference and interpretations. Therefore, we chose to focus on longitudinal studies for the charted review to better understand temporal and potential causal relations between wealth and depression. We present findings on the full review, which included both cross‐sectional and longitudinal studies, and the charted review, which provided summarized information about the longitudinal studies.

## 3 RESULTS

### 3.1 Summary of full review findings

#### 3.1.1 Full review characteristics

**FIGURE 2:**
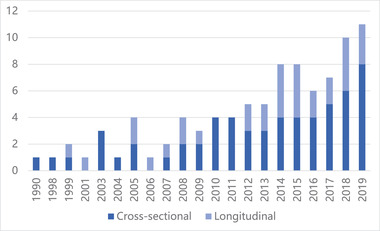
Number of articles published on wealth and depression by study design

We identified 96 published articles on wealth and depression through July 2020 (Appendix SB). Table 1 shows the characteristics of the articles in the full review. The number of such articles published increased over time. The first article included in the review was published in 1990 (*n* = 1). In 2019, the last full year included in the review, 11 articles on wealth and depression were published. Figure 2 shows the distribution of articles published over time through 2019 by study design. The majority of articles (*n* = 86, 90%) featured study populations that were ages 18 years and older. Among those studies, 33 (34%) included only adults ages 50 years or older. The majority of studies (*n* = 64, 67%) used a cross‐sectional study design, measuring one point in time. The most commonly used measure of depression was the Center for Epidemiological Studies‐Depression (CES‐D) (*n* = 34, 35%). The second most commonly used measure of depression was the Patient Health Questionnaire‐9 (PHQ‐9) (*n* = 7, 7%), followed by Kessler scales (*n* = 6, 6%) and then the Children's Depression Inventory (CDI) (*n* = 5, 5%). The “Other,” category represents measurements of depression that were not clinically validated or used in other studies (*n* = 11, 11%). Measures of depression used in fewer than five studies are listed in Table 1. The country featured most frequently in studies about wealth and depression was the United States (*n* = 30, 31%), followed by multiple country comparisons (*n* = 12, 13%) and the United Kingdom (*n* = 11, 12%).

**TABLE 1:** Characteristics of articles on wealth and depression included in full review (*n* = 96)

Characteristics	Frequency (*N*)	Percentage (%)
Age of study population		
All adults	86	90
Adults, mix (≥18 years)	53	55
Adults, older only (≥50 years)	33	34
Youth (<18 years)	8	8
Youth and adults	2	2
Study design		
Cross‐sectional	64	67
Longitudinal	32	33
Definition of depression		
CES‐D	34	35
Other	11	11
PHQ‐9	7	7
Kessler	6	6
CDI	5	5
Used in fewer than five studies^†^	33	34
Country		
Unites States	30	31
Multiple	12	13
United Kingdom	11	12
Uganda	6	6
Mexico	4	4
India	3	3
South Africa	2	2
Serbia	2	2
Tanzania	2	2
South Africa	2	2
Ghana	2	2
Pakistan	2	2
Vietnam	2	2
Other^‡^	16	17
Relation between wealth and depression		
Inversely	56	58
Complicated	31	32
Not significant	6	7
Directly	3	3

*Note*: Percentages may not add up to 100% due to rounding.

Abbreviations: CDI, Children's Depression Inventory; CES‐D, Center for Epidemiological Studies Depression; PHQ‐9, Patient Health Questionnaire‐9.

^†^Measurements of depression used in fewer than five studies: GHQ (*n* = 3), ICD‐10 (*n* = 3), CIDI (*n* = 4), Euro‐D (*n* = 3), HSCL (*n* = 2), DSM (*n *= 2), EPDS (*n* = 2), Beck (*n* = 2), SCL‐90 (*n* = 1), ZLDSI (*n* = 1), WHODAS–II (*n* = 1), HSCL‐25 (*n* = 1), CES‐D and K‐6, (*n* = 1), CES‐D and Euro‐D (*n* = 1), DASS‐21 (*n *= 1), SRQ‐20 (*n* = 1), DSRS (*n* = 1), MHI (*n* = 1), and did not say (*n* = 1). “Other” depression describes measurement that did not use a validated instrument.

^‡^“Other” countries (*n* = 1) included Korea, Thailand, Sweden, Ethiopia, Denmark, New Zealand, Hong Kong, Haiti, Mozambique, Peru, Czech Republic, Philippines, Kenya, Nepal, China, and Netherlands.

#### 3.1.2 Measurement of key constructs

Depression was defined in a relatively standard way across studies, with the CES‐D being the most commonly used measure for screening depressive symptoms. Wealth, on the other hand, was defined using a broad range of measures. In certain cases, wealth was defined as the absence of poverty. For example, Karimli et al. (2019) created a childhood poverty index which included owning more than two changes of clothes, owning a blanket, having eaten meat or fish at least once in the last week, having eaten three meals per day in the last 7 days, and having at least two pairs of shoes. Others measured access to banking institutions, such as owning a checking account, savings account, or credit card (Yoshikawa et al., 2008). In other cases, wealth was defined as the summation of financial assets such as total amount of funds in checking and savings accounts, certificates of deposit, Treasury bills, bonds, Individual Retirement Accounts (IRAs), stocks, mutual funds, real estate (besides housing), vehicles, businesses, other savings and assets, and minus debts (McInerney et al., 2013). Thus, the meaning and operationalization of wealth varied greatly from study to study.

#### 3.1.3 Full review findings on relation between wealth and depression

The majority of studies in the full review (*n* = 56, 58%) found an inverse relation between greater wealth and depression. Thirty‐one articles (32%) reported complicated findings, showing multiple associations in potentially different directions among subgroups. Six articles (7%) reported nonsignificant associations between wealth and depression. Three articles (3%) reported a direct relation between wealth and depression; thus, these three articles showed that more wealth was associated with more depression.

### 3.2 Summary of charted review findings

#### 3.2.1 Charted review characteristics

Table 2 shows the characteristics of the charted review. We identified 32 longitudinal articles about wealth and depression published through July 2020. Similar to the full review, the majority of articles (*n* = 27, 84%) were adult study populations. Among adult population articles, 12 (38%) featured only older adults, ages 50 years or older. Five articles (16%) used pediatric study populations. We identified 25 articles (78%) that used observational study designs and seven articles (22%) that used experimental intervention designs, all of which were randomized control trials with randomly selected treatment and control groups. The most commonly used measure of depression among the charted review articles was the CES‐D (*N* = 16 articles, 50%), which represented a higher percentage of use among articles than the full review (35%). The Kessler scale and the CDI) were each used in four articles (13% each). The remaining definitions of depression used are included in Table 2. The United States represented a larger fraction of studies in the charted review (*n* = 18, 56%) relative to studies in the full review (31%). The most commonly used data source for the longitudinal studies on wealth and depression was the Health and Retirement Study (HRS), featured in nine of the 32 (28%) longitudinal studies (Table S1). Three longitudinal studies used the Panel Study of Income Dynamics (PSID). Two longitudinal studies included used the English Longitudinal Study of Ageing (ELSA) and National Survey of Families and Households (NSFH). Twenty‐three of the 32 (72%) longitudinal studies used data from population representative standing panels monitoring health. Nine (28%) used data collected specifically for the purpose of the study (including all seven of the experimental studies and two observational studies).

**TABLE 2:** Characteristics of longitudinal studies (*n* = 32)

Characteristics	Frequency (*N*)	Percent (%)
Age of study population		
All adults	27	84
Adults, mix (≥18 years)	15	47
Adults, older only (≥50 years)	12	38
Youth (<18 years)	5	16
Study design		
Observational	25	78
Experimental	7	22
Definition of depression		
CES‐D	16	50
Kessler	4	13
CDI	4	13
Other	2	6
Proxy report	1	3
PHQ‐9	1	3
SCL	1	3
CES‐D, Euro‐D	1	3
CES‐D, Kessler	1	3
GHQ	1	3
Country		
United States	18	56
Uganda	3	9
United Kingdom	3	9
Multiple	2	6
Sweden	1	3
Columbia	1	3
Czech Republic	1	3
Ghana	1	3
Netherlands	1	3
New Zealand	1	3
Relation between wealth and depression		
Inversely	16	50
Complicated	12	38
Not significant	3	9
Directly	1	3

Abbreviations: CDI, Children's Depression Inventory; CES‐D, Center for Epidemiological Studies Depression; Euro‐D, European Union Depression scale; GHQ, General Health Questionnaire; PHQ‐9, Patient Health Questionnaire‐9; SCL, The Symptom Checklist‐core.

#### 3.2.2 Charted review findings on relation between wealth and depression

Half of the studies (*n* = 16, 50%) in the charted review reported an inverse relation between wealth and depression. Twelve studies (38%) reported complicated findings. Three studies (9%) reported nonsignificant associations. One study (3%) reported a direct relation between wealth and depression, where depression increased as wealth increased. All but one article (*n* = 31, 97%) examined the relation between baseline wealth and subsequent depression (finding in more than half of articles that greater baseline wealth was associated with less subsequent depression); one article (*n* = 1, 3%) found that greater baseline depression was associated with less subsequent wealth. Thus, all but one article assessed how wealth predicted depression; one article estimated how depression predicted wealth.

#### 3.2.3 Charted review themes

Table 3 shows a charted review of findings from the longitudinal studies on wealth and depression. Studies assessing depression and wealth often used changes in wealth as their analytic focal point, either following a decrease due to an economic recession or an increase due to experimental savings initiatives. Studies could generally be grouped into five themes: observational studies following macro‐economic events (*n* = 7); observational studies measuring trends in depression across the lifecourse (*n* = 7); observational studies following job loss (*n* = 4); observational studies measuring general patterns between wealth and depression (*n* = 7); and experimental studies implementing a specific savings intervention (*n* = 7). We report findings by these themes.

**TABLE 3:** Charted data of longitudinal studies on wealth and depression (*n* = 32)

References	Research question	Study population	Study design	Definition of depression	Did the study define how wealth was measured?	Was wealth associated with less depression?
Rodriguez et al. (1999)	How do risk factors for depression following unemployment vary by white and black adults?	Adults ages 17– 65 years from the National Survey of Families and Households (NSFH). Wave 1 (1987); Wave 2 (1992–1993). *N* = 8029 (*n* = 1369 African‐American and *n* = 6660 white persons). USA.	Observational prospective cohort study on nationally representative panel data.	Past week depressive symptoms. Center for Epidemiological Studies’ Depression Scale (CES‐D) 15‐item scale. Continuous measure. Log transformed.	No. The authors state, “Wealth indicated by total assets.”	Complicated. Wealth was predictive of depression at Time 2 (5–6 years after baseline) for white persons but not for African‐American persons.
Hraba et al. (2001)	How does age relate to changes in depression and behavior?	Adults ages 20–80 years from a random sample of Czech Republic households. Wave 1 (1994) *n* = 593 men and *n* = 721 women; Wave 2 (1995) *n* = 562 men and *n* = 686 women; Wave 3 (1996) *n* = 538 men and *n* = 656 women; Wave 4 (1999) *n* = 497 men and *n* = 603 women. Czech Republic.	Observational prospective cohort study on nationally representative panel data	Past week depressive symptoms. Derogatis's Symptom Checklist (SCL‐90) 12‐item scale.	Yes. Measured wealth in 1995 and change in wealth between 1989 and 1995. Respondents were asked, “Imagine you were completely insured, and all your property (house, cottage, car, equipment, personal belongings, etc.) was lost in a natural disaster. What would be the amount of money that would need to be paid to make up for this loss if the disaster occurred today?” The answers were categorized into nine income levels (1 = less than 50,000 Crowns, 9 = more than 2,000,000 Crowns).	Complicated. An increase in wealth between 1995 and 1989 was associated with a reduction in depression between 1995 and 1999 for women but was not associated with a significant change in depression for men.
Silveira et al. (2005)	What mutable factors such as wealth or caregiver status are related to symptom burden for older adults at the end of life?	Deceased older adults who were 70 years or older when they died from the Health and Retirement Survey (HRS). *N* = 2604. USA.	Observational retrospective cohort study on nationally representative panel data. Proxies reported on patents’ symptom burdens in the time between the participants’ last interview and end of life within the year after study participants’ death.	Proxy report of 1‐month patient depression in the year before death. For time before death, proxies were asked, "Was there a period of at least 1 month during the last year of life when he had (this symptom)?”	No. Study used net worth categories of less than $10,400; $10,400–70,000; $70,001–182,000; and more than $182,000. While other studies using the HRS have defined net worth, this study did not expand on how the variable was defined.	Not significant. Wealth was associated with less depression in a dose‐response relation but it was not statistically significant.
Smith et al. (2005)	Does wealth protect well‐being following the onset of disability?	Older adults ages 50–61 years from the Health and Retirement Survey (HRS). Wave 1 (1992) *N* = 12,521 through Wave 5 (2000) *N* = 16,765. USA.	Observational prospective cohort study on nationally representative panel data.	Subjective well‐being. Adapted four items from the CES‐D depression inventory. Responses to questions on whether participants were happy, enjoyed life, were sad (reverse‐scored), and were lonely (reverse‐scored) were averaged.	Yes. Total household assets defined as stocks, bonds, savings, housing equity, and other assets, subtracting current debts.	Yes. Persons with above median wealth at baseline had less depression following new disability than persons with below median wealth. Over time, the well‐being of persons with below median wealth improved, leading to similar well‐being between the two groups.
Gallo et al. (2006)	Is involuntary job loss among older adults associated with depression over time, and do displaced low‐SES adults show greater symptoms of long‐term mental scarring following job loss?	Older adults ages 51−61 years from the Health and Retirement Survey (HRS). Wave 1 (1992) *N* = 12,521; Wave 2 (1994) *N* = 11,596; Wave 3 (1996) *N* = 11,200; Wave 4 (1998) *N* = 10,8565. For this analysis, adults had to be at risk for job loss in Wave 1 of the HRS (as in, they had to work for pay.) *N* = 3555. USA.	Observational prospective cohort study on nationally representative panel data.	Past week depressive symptoms. Center for Epidemiological Studies’ Depression Scale (CES‐D) eight‐item scale. HRS changed the way it asked about depressive symptoms between 1992 and later waves (for ease of reporting symptoms over phone interviews). Linking algorithm used to generate comparable depressive symptom scores across waves, citing other studies.	Yes. Net worth calculated by totaling household nonhousing asset amounts recorded in Wave 1 (1992), including 1992 values of savings and checking accounts, certificates of deposits, bonds, Treasury bills, individual retirement accounts, stocks and mutual funds, vehicles, business equity, equity in real estate other than primary residence, and other reported nonhousing assets. Authors dichotomized net worth as greater than $38,000 (the median). Changes in assets over time were not measured.	Yes. At Wave 3 (4‐year), authors reported a statistically significant weaker effect of job loss on depressive symptoms among those with above to below median nonhousing net worth. Participants with below median net worth reported increased depressive symptoms following job loss while participants with above median net worth reported no difference in depressive symptoms compared to persons who had not lost jobs. Results were the same at Wave 4 (6‐year) follow‐up.
Dew (2007)	How do assets and consumer debt relate to marital outcomes?	Adults in the National Study of Families and Households (NSFH) married at Wave 1 (1987) through Wave 2 (4–7 years later). *N* = 3731. USA.	Observational prospective cohort study on nationally representative panel data.	Past week depressive symptoms. Center for Epidemiological Studies’ Depression Scale (CES‐D) 12‐item scale.	Yes. Net asset value at Wave 1 defined by savings, home equity, and investments. Authors used log transformation (base 10) for assets to correct for skewness. Changes in assets over time were not measured.	Complicated. While assets were significantly negatively correlated with depression in the bivariable model, they were no longer predictive in the structural equation model (accounting for other factors). Thus, wealth and depressive symptoms were inversely associated, but the relation was mediated by other factors. Debts, however, negatively predicted depression.
Mossakowski (2008)	Are racial/ethnic mental health disparities explained by socioeconomic inequality experienced earlier in the life course?	Young adults ages 27–35 years from the National Longitudinal Survey of Youth (NLSY). Wave 1 (1979) through Wave 13 (1992). *N* = 7975. United States.	Observational prospective cohort study on nationally representative panel data.	Past week depressive symptoms. Center for Epidemiological Studies’ Depression Scale (CES‐D) 20‐item scale. Measured in 1992.	Yes. Wealth was measured with homeownership and net worth. Participants were asked, “If you and your spouse/partner were to sell all your major possessions (including your home), turn all of your investments and other assets into cash, and pay all of your debts, would you have money left over, break even, or be in debt?” Three dichotomous indicator variables were created: “Break even” for zero net worth, “in debt” for negative net worth, and “have money left over” for positive net worth.	Yes. Having zero or negative net worth relative to positive net worth was associated with greater depressive symptoms for young adults in the study (adjusting for family background, race, age, and marriage). Wealth also partially mediated the effect of race and ethnicity on depression. Duration of childhood poverty significantly predicted depressive symptoms for adults ages 27–35 years.
Yoshikawa et al. (2008)	How does access to institutional resources relate to economic hardship and psychological distress among immigrant families?	Female adults who had given birth in three large NYC hospitals as part of the Early Childhood Cohort (ECC) of the Center for Research on Culture, Development and Education (CRCDE) Wave 1 (2004–2005). Multiple waves (14 months and 24 months later). *N* = 324. USA.	Observational prospective cohort study on a group of NYC immigrant mothers and their children.	Past 30‐day psychological distress. Kessler‐6 scale. Measured at the 14‐month and 16‐month waves.	Yes. Institutional resource access was defined by having household access to (1) a checking account, (2) a savings account, (3) a credit card, or (4) a driver's license. Responses to these items were summed to create an index (0–4). Collected at the 14‐month wave.	Not significant. Institutional resource access at 14 months was associated with less financial hardship, which was significantly related with psychological distress. However, there did not appear to be a significant relation between institutional resource access and psychological distress.
Carter et al. (2009)	Is asset wealth associated with mental health, and are the associations independent of other socioeconomic factors and prior health status?	Adults ages 25 years or older from the Survey of Families, Income and Employment (SoFIE) conducted in New Zealand Wave 1 (2002) through Wave 3 (2004). *N* = 15,340. New Zealand.	Observational prospective cohort study on nationally representative panel data.	Past 30‐day psychological distress. Kessler‐10 scale. Measured at Wave 3 (2004).	Yes. Net worth calculated by subtracting the total value of all liabilities from the total value of all assets for couples and individuals. Overall wealth calculated by taking a couple's total wealth and dividing it by two if a participant was in a couple; otherwise, individual wealth was used. Wealth quintiles defined by (Q1) < $25,587; (Q2) $25,587–70,313; (Q3) $70,313–128,087; (Q4) $128,087– < 232,932; and (Q5) $232,932 and above.	Yes. Bivariable models showed an inverse, linear relationship between wealth and psychological distress. Adjusted univariable models showed an increase in odds of psychological distress in the lowest relative to the highest wealth quintile. The odds radio for psychological distress was 1.73 for low relative to high wealth quintiles when controlling for age, sex, demographic characteristics, and socioeconomic factors; the odds ratio was 1.45 when additionally controlling for health status.
Ssewamala et al. (2012)	Can a comprehensive microfinance intervention reduce depression among AIDS‐orphaned children?	Youth who had lost one or both parents due to AIDS. Wave 1 (2005) Wave 2 (10–12 months after baseline) Wave 3 (20–24 months after baseline). *N* = 262. Uganda.	Experimental study: randomized control trial with microfinance intervention on a group of AIDS‐orphaned children.	Past 2‐week depressive symptoms. Children's Depression Inventory (CDI) 10‐item scale.	Yes. Economic assets defined by microfinance intervention. The Suubi‐Maka intervention had three components: (1) a matched savings account in the child's name. The program matched 2:1 for every dollar contributed, up to $10/month, (2) classes on financial management and business development, (3) adult mentorship that encouraged to youth to save and that provided small business startup guidance.	Yes. There were significant differences in depression between the control group and treatment group at Waves 2 and 3. The Suubi intervention group reported significantly lower depression over time (meanwhile the rate of depression did not change over time for the control group).
Berchick et al. (2012)	How does the association between involuntary job loss and depressive symptoms vary based on five features of socioeconomic status: education, income, occupational prestige, wealth, and homeownership?	Adults ages 25 years or older from the Americans’ Changing Lives (ACL) survey across wave pairs Waves 1 and 2 (1986−1989), Waves 2 and 3 (1989−1994), Waves 3 and 4 (1994−2002). *N* = 1510. USA.	Observational prospective study using panel data.	Past week depressive symptoms. Center for Epidemiological Studies’ Depression Scale (CES‐D) 11‐item scale. Previous wave depression symptoms controlled for to account for differences at baseline.	Unclear. Wealth defined as a dichotomous measure of financial assets (less than $50,000, equal to or above $50,000) and homeownership. Component definition of what was included in financial assets not included in this study.	Not significant. Wealth, income, and homeownership were not related to reduced depressive symptoms following unexpected job loss. Higher education was related with lower risk of depression while occupational prestige was related with a higher risk of depression following job loss.
McInerney et al. (2013)	What is the effect of a large, sudden loss of wealth on depression?	Older adults from the Health and Retirement Survey (HRS). Wave 1 (2006) and Wave 2 (2008) *N* = 10,020. USA.	Observational prospective cohort study on nationally representative panel data following the 2008 stock market crash.	Past week depressive symptoms. Center for Epidemiological Studies’ Depression Scale (CES‐D) eight‐item scale. Negative symptoms summed and positive symptoms were reverse coded for a cumulative depressive symptom index. A binary indicator for CES‐D scores of 3 or higher was created and a binary score for participants who reported regularly taking medicine to relieve depression or anxiety. Subjective, nonclinically validated measures of depression also used, including a binary indicator of 1 if participants reported “feeling depressed” in the past week, and 0 if not.	Yes. Nonhousing wealth measured in 2008 dollars defined as total nonhousing assets minus debt; nonhousing assets included the amount of money in checking and savings accounts, certificates of deposit, savings bonds and Treasury bills, bonds, total Individual Retirement Accounts, stocks, mutual funds, real estate other than housing, vehicles, businesses, plus other savings and assets.	Complicated. For clinically validated measures of depression: participants owning no stocks or IRA accounts were not affected by the October 2008 stock market crash. Among stock holders, wealthier participants reported more depressive symptoms after than before the crash; however, this effect was not statistically significant. Below median wealth stock holders reported significantly fewer depressive symptoms after the crash than before it. Antidepressant use increased following the crash among high wealth stock holders. For subjective report of health status, the wealthier half of stockholders were more likely to report feeling depressed following the crash than before it.
Han et al. (2013)	Can a family economic empowerment program impact the depression and hopelessness of AIDS‐orphaned children?	Youth ages 12–14 years who had lost one or both parents due to AIDS. Wave 1 (2005) Wave 2 (10–12 months after baseline). *N* = 270. Uganda.	Experimental study: randomized control trial with microfinance intervention on a group of AIDS‐orphaned children.	Past 2‐week depressive symptoms. Children's Depression Inventory (CDI) 10‐item scale.	Yes. Economic assets defined by microfinance intervention. The Suubi‐Maka intervention had three components: (1) a matched savings account in the child's name. The program matched 2:1 for every dollar contributed, up to $10/month, (2) classes on financial management and business development, and (3) adult mentorship that encouraged to youth to save and that provided small business startup guidance.	Yes. Controlling for socioeconomic factors for children and their guardians, children in the treatment group reported decreased depressive scores at Wave 2. The gender of the child's guardian was predictive of a child's depressive symptoms. For example, children with female guardians were more likely to report depressive symptoms at Wave 2. Having an older guardian was also associated with higher depression level. Physical health was significantly related with mental health; children reporting good or excellent health reported lower depressive symptoms.
Hamoudi and Dowd (2014)	How did the rise in housing prices from the mid‐1990s to the mid‐2000s impact psychological and cognitive outcomes for older Americans?	Older adults from the Health and Retirement Survey (HRS) born between 1924 and 1960. Baseline Wave 1 Group 1 (1992), Baseline Wave 1 Group 2, and Follow‐up Wave 2 (2006) *N* = 4207. USA.	Observational prospective cohort study on nationally representative data combined with DataQuick home value data.	Past week depressive symptoms. Center for Epidemiological Studies’ Depression Scale (CES‐D) eight‐item scale.	Yes. Wealth defined by: home value at baseline, logged and splined), estimated by DataQuick; dollar value of nonhousing wealth at baseline (logged and splined); share of housing equity at baseline (housing debt; indicator measured equity stake as less than two thirds of purchase value and less than full purchase value of the house); nonhousing debt at baseline (with indicators as none, more than one fifth, or more than three quarters of total nonhousing wealth).	Yes. Among home owners, steeper growth in housing value was associated with improved psychological health. Rising prices were less beneficial for psychological health for home renters than for home owners.
Riumallo‐Herl et al. (2014)	Does late career job loss increase depression among older workers in the United States and Europe? Does job loss have a greater effect on persons in the United States, and particularly those with low wealth, than persons in Europe?	Older adults ages 50–64 years who were employed in the 2004 or 2006 from the Health and Retirement Survey (HRS) and the Survey of Health, Ageing and Retirement in Europe (SHARE). Wave 1 (2004), Wave 2 (2006), Wave 3 (2010). *N* = 15,055. United States. Europe: Austria, Belgium, Czech Republic, Denmark, France, Germany, Greece, Italy, The Netherlands, Poland, Spain, Sweden, and Switzerland.	Observational prospective cohort study on nationally representative panel data.	Past week depressive symptoms (USA) and past month depressive symptoms (Europe). Center for Epidemiological Studies’ Depression Scale (CES‐D) eight‐item scale (USA, HRS). Euro‐D 12‐item scale (Europe, SHARE). Authors harmonized the scales to compare.	Yes. Wealth defined as the sum of all household financial and housing wealth minus debts. Missing values imputed using hot‐deck procedures. Household wealth and income were divided by the square root of household size. Authors transformed wealth values into 2006 US dollars using power parity adjustments. The natural log of income and wealth were used to reduce the role of outliers and to account for nonlinearities.	Complicated. The relation between wealth and depression was stronger in the United States in Europe. Job loss increased depressive scores in both samples, although the effect on depressive scores was greater in the United States (4.78% [95%CI 0.823%−8.74]) than in Europe (3.35% [95%CI 0.486%−6.22%]). The effect of job loss on depressive scores was weaker for adults with wealth before the job less in the United States than among adults with little or no wealth; there was no interaction between job loss and wealth on depression among European adults.
Cagney et al. (2014)	Are neighborhood‐level foreclosure rates associated with onset of depressive symptoms among older adults?	Older adults ages 57 years and older from the National Social Life, Health, and Aging Project (NSHAP) survey. Wave 1 (2005–2006); Wave 2 (2010–2011). *N* = 2261. USA.	Observational prospective cohort study on nationally representative panel data, combined with geocoded data linked to the American Community Survey (ACS).	Past week depressive symptoms. Center for Epidemiological Studies’ Depression Scale (CES‐D) 11‐item scale, called the “Iowa Short Form.” A dichotomous variable at 8 (range 0–22) was used.	Yes. Respondents were asked to report the value of assets held in homes, cars, rental properties, businesses, savings, stocks, mutual funds, and pensions, over and above their loans.	Complicated. There was no significant association between increased assets and depression among older adults across the foreclosure process (notices of default, auctions, or real‐estate owned). Living in an area with an increase in housing foreclosure was associated with a significant increase in depressive symptoms.
Huang et al. (2014)	Does universal asset building at birth for children influence their mother's mental health?	Female adults who had given birth in Oklahoma in 2007 as part of the SEED for Oklahoma Kids (SEED OK), a randomized policy experiment of Child Development Accounts (CDAs). Wave 1 (2008) *N* = 2704, Wave 2 (2011) *N* = 2272. Final sample: *N* = 2223. USA.	Experimental study: randomized control trial with Child Development Accounts (CDAs) savings intervention for children born in 2007, weighted to be representative of all births in Oklahoma that year.	Past week depressive symptoms for mothers. Center for Epidemiological Studies’ Depression Scale (CES‐D) four‐item scale.	Yes. The SEED OK experiment offered three financial incentives to mothers in the treatment conditions. (1) The state deposited $1000 of SEED OK funds into a state‐owned OK 529 account opened automatically for children in the treatment group in 2008. (2) Participants were encouraged to open and contribute to their own participant‐owned OK 529 accounts. The program offered to make the $100 initial contribution needed to open accounts for treatment participants before April 2009. (3) Low‐ and middle‐income treatment groups were eligible for matching deposits based on level of income. Two savings indicators used to test for mediation: (1) a binary indicator for whether mothers owned an OK 529 account for their children by the end of 2011 (yes or no) and (2) a binary indicator if a deposit had been made into these accounts (yes or no).	Yes. Mean scores of depressive symptoms were significantly lower among mothers in the treatment arm than the control arm. The effect of the treatment on mothers was significantly larger on the low‐income and low‐education subsamples than for the whole sample. Two savings variables were used to test for mediation; the two savings variables were negatively associated with CES‐D but neither association was statistically significant (potentially due to the small number of women who contributed to savings accounts). The two savings indicators did not mediate the relation between the OK SEED intervention and depressive symptoms.
McGovern and Nazroo (2015)	Are class‐related lifestyle characteristics important for health in later life? Is wealth linked to health status directly through material benefits and indirectly through subjective social status (and access to cultural goods such as going to the theater)?	Older adults ages 50 and over in the English Longitudinal Study of Ageing (ELSA) study. Wave 1 (2002−2003); through Wave 5 used. *N* = 6241. UK.	Observational prospective cohort study on nationally representative panel data.	Past week depressive symptoms. Center for Epidemiological Studies’ Depression Scale (CES‐D) eight‐item scale.	Yes. Economic capital was defined by total nonpension wealth for the individual, including financial assets, property, other physical assets and the assets of any business they owned and was measured, subtracting any outstanding debts. The wealth variable was scaled using £100,000 units. Cultural capital measured by frequency of visits to museums and galleries theaters, opera, and the cinema. Answers categorized along a six‐point scale ranging from never to two times per month or more. Social capital was measured through number of close relationships, committee membership, and frequency of volunteering. Subjective social status (SSS) measured using the MacArthur scale, a 10‐rung ladder.	Yes. Wealth had a small, direct effect on depression; wealth reduced the number of depressive symptoms a participant reported. Objective social class (including wealth) was partially mediated by subjective social status. Class‐based inequities existed among older persons who were retired.
Hounkpatin et al. (2015)	Is income or wealth a stronger predictor of depressive symptoms, and does social comparison or rank matter more than absolute value for mental health?	Older adults who had graduated from high school in 1957 in the Wisconsin Longitudinal Study (WLS) and older adults ages 50 years and over in the English Longitudinal Study of Ageing (ELSA) study. Wave 1 (WLS, 1992) (ELSA, 2002, *N* = 11,264), Wave 2 (WLS, 2003, *N* = 6494) (ELSA, 2008, *N* = 6425). USA and UK.	Observational prospective cohort study on panel data.	Past week depressive symptoms. Center for Epidemiological Studies’ Depression Scale (CES‐D). Featured in both studies. A continuous measure was used.	Unclear. Authors used relative rank and absolute rank of income and “total wealth.” For absolute measures, in WLS, household income (in dollars) was used. In ELSA, net wealth (in British pounds) was used. For relative measures, a relative rank measure was calculated for each participant based on the person's wealth within the reference group (the ranked position of the person's wealth within the reference group for ELSA.) Absolute wealth and transformed deviation of absolute wealth from mean wealth within the reference group were used.	Complicated. Wealth more strongly predicted depressive symptoms than income in the ELSA but not in the HRS. Results supported the wealth rank hypothesis.
Karasz et al. (2015)	Can the Action to Improve Self‐esteem and Health through Asset building (ASHA) intervention program reduce depression and improve financial independent for South Asian women immigrants in NYC?	Female adults ages 18–70 years who were Bangladeshi immigrants living in NYC with income at 200% of the federal poverty level or lower and with a PHQ‐9 score of 8 or above. Wave 1 (2012–2013) Wave 2 (26 weeks later). *N* = 66. USA.	Experimental study: randomized control trial with asset intervention on a group of South Asian immigrant women in NYC	Past 2‐week depressive symptoms. Patient Health Questionnaire (PHQ‐9) 9‐item scale. South Asian Tension 23‐item Scale also used to measure indigenous distress.	Yes. The study highlighted three assets: psychological skills, social networks, and financial assets. The Action to Improve Self‐esteem and Health through Asset building (ASHA) was a 26‐week treatment intervention. At the end of the 26‐week intervention, participants could elect to receive a triple match. Participants could withdraw funds for purchases that would foster financial independence, i.e., training programs, education, and business development, etc.	Yes. Depressive symptom severity was significantly lower after the intervention for the treatment group. There was a slight but nonsignificant reduction in depressive symptom severity for women in the control group at Time 2. The difference in mean PHQ‐9 scores between the control and intervention group at Time 2 was significant (*p* = .02). Women in the treatment group used the matched financial assets to start businesses, take English language courses, gain drivers licenses, and take other courses.
Yilmazer et al. (2015)	How did declines in housing wealth following the global economic recession of 2008 affect depression among homeowners?	Adults in the Panel Study of Income Dynamics (PSID). Wave 1 (2007), Wave 2 (2009), Wave 3 (2011). *N* = 4007. USA.	Observational prospective cohort study on nationally representative panel data.	Past 30‐day psychological distress. Kessler‐6 scale. The following outcomes were measured: (1) psychological distress (sum of responses to Kessler‐6 scale), (2) feeling depressed (score of 13 or greater on the Kessler scale), (3) clinical diagnosis of depression, and (4) behavioral consequences of stress (measured by interference with life or activities and number of days unable to work or carry out normal activities due to distress). Note: depression measured for the head of the household (the husband if a couple).	Yes. Nonhousing wealth defined as the sum of assets (excluding primary residence) subtracting all debts (excluding mortgages). Housing wealth defined as the difference between value of a primary residence and remaining mortgage. Net wealth defined as the sum of nonhousing wealth and housing wealth. Additionally, homeowners were asked if they had difficulty with their mortgages and if the foreclosure process had started. Note: wealth measured at the household level.	Yes. Decreases in housing and nonhousing wealth were each associated with increased psychological distress. As the ratio of housing to nonhousing wealth decreased, psychological distress and feeling depressed increased. Households reporting difficulties paying their mortgage or reporting foreclosure had significantly higher levels of psychological distress, feeling depressed, and depression diagnosis. Difficulties paying mortgage and experiencing foreclosure had more severe effects on psychological distress and on life interferences than did the declines in housing or nonhousing wealth.
Wilkinson (2016)	Did the Great Recession contribute to worsened anxiety and depression over a 4‐year period among older adults? Did objective or subjective financial indicators predict worsening mental health between 2006 and 2010? Did objective financial indicators influence financial strain? If so, did objective indicators and subjective indicators each effect mental health?	Older adults age 51 years and older in the Health and Retirement Survey (HRS). Wave 1 (2006) and Wave 2 (2010) *N* = 5366. USA.	Observational prospective cohort study on nationally representative panel data.	Past week depressive symptoms. Center for Epidemiological Studies’ Depression Scale (CES‐D) eight‐item scale. Items were summed for a continuous count of depressive symptoms.	Yes. The study measured subjective and objective indicators of financial situation. Objective indicators included: financial wealth, net home equity, household income, and labor force participation. Financial wealth defined by the sum of all nonhousing assets, including stocks, bonds, and bank accounts subtracting any debts. Net home equity defined by the value of primary residence subtracting any mortgages or home loans. Financial wealth, net home equity, and household income were coded in thousands of dollars and log‐transformed. Subjective indicators included financial strain. Financial strain defined by responses to questions about difficulty meeting monthly payments and about satisfaction with one's present financial situation.	Complicated. Objective financial measures had little effect on mental health. The subjective indicator, financial strain, was a significant predictor of worsened mental health. Objective financial resources (financial wealth, net home equity, and household income) all decreased on average from 2006 to 2010.
Lê‐Scherban et al. (2016)	Is greater family wealth during childhood associated with better mental health in young adulthood? Does education mediate the relation between parental wealth and young adult mental health?	Young adults ages 18–27 years from the Transition into Adulthood Study (TAS) supplement to the Panel Study of Income Dynamics (PSID). Pooled data from 2005 to 2011. *N* = 2060. USA.	Observational prospective cohort study using pooled nationally representative panel data.	Past 30‐day psychological distress. Kessler‐6 scale. Low (0–4), moderate (5–12), and serious (13–24) psychological distress reported.	Yes. Net household wealth defined by household head's report of business equity, bank accounts, money market funds, certificates of deposit, government savings bonds, treasury bills, real estate equity, stocks, vehicle equity, individual retirement accounts (IRAs), other assets (such as life insurance policy, rights in a trust or estate), and other debts. Wealth measures were converted to 2010 dollars using the Consumer Price Index. Authors created childhood average household wealth measures by taking the average of all available wealth measurements for participants starting in the year of his/her birth until their 18th birthday. Note: childhood average household wealth was extremely skewed (with a range of $190,000 to $24,800,000 and a median of $19,900) so an average childhood wealth percentile based on the distribution of wealth was created.	Complicated. Young adults in the highest quintile for family wealth during childhood had significantly lower odds of serious psychological symptoms than persons in the below‐median wealth quintiles. The relation between family wealth and psychological distress was not statistically significant for mild and moderate psychological distress. The relation between greater family wealth in childhood and lower serious psychological distress was not mitigated by mother's education; it was weakened by family income percentile. Greater childhood wealth was related to a higher prevalence of serious psychological distress among persons without a high school degree but lower prevalence of distress among persons with more education.
Nieuwenhuis et al. (2017)	Does moving to a new neighborhood context with a different level of wealth change mental health among adolescents? Does this relation vary by family income level?	Youth ages 12–20 years from the Conflict and Management of Relationships (Conamore) panel. Wave 1 (2001–2002), *N* = 1313, through Wave 5 (2005–2006) *N* = 1275 used. Final analytic sample: *N* = 926. The Netherlands.	Observational prospective cohort study on nationally representative panel data combined with postcode area characteristics from Statistics Netherlands and population register data from the Statistics Netherlands System of Social Statistical Datasets (SSD).	Past 2‐week depressive symptoms. Children's Depression Inventory (CDI) 27‐item scale. Authors created a dichotomous variable such that 30% of the sample was defined as depressed.	Yes. Neighborhood wealth defined by average property value in postcode in 2004 according to the Statistics Netherlands data. Family income defined by adding the income of the two highest earners in a household according to the Statistics Netherlands register data. The income variable was standardized.	No. Relocating to a more affluent neighborhood was associated with an increase in depression among adolescents. Income did not change the effect. No gender differences in depression by neighborhood wealth were reported. Adolescent girls were more prone to depression than boys, but the interaction with neighborhood wealth and gender on depression was not significant.
Pool et al. (2017)	Are wealth shocks experienced late in life associated with elevated depressive symptoms and elevated cost‐related medication nonadherence?	Older adults ages 51−64 years in the Health and Retirement Survey (HRS). Wave 1 (1992) through Wave 11 (2012). Final analytic sample: *N* = 19,2816. USA.	Observational case–control study: nested cross‐over approach, a within‐person design, on national panel data. Measured depression at time of wealth shock and two waves later (for 1 wave induction period).	Past week depressive symptoms. Center for Epidemiological Studies’ Depression Scale (CES‐D) eight‐item scale. Elevated depressive symptoms defined as a dichotomous variable by reporting experiencing three or more of the indicators for most/all of the time in the past week.	Yes. Net worth defined by the sum of all assets, including real estate, business assets, vehicle worth, stocks, bank account values, and individual retirement accounts minus all debts, including mortgage, home equity loans, and unsecured debts. A negative wealth shock was defined as a negative change in net worth of 75% of more between two consequence waves. Negative wealth shock dichotomized at each wave. Participants considered “at risk” for wealth shock until the first occurrence. Note: authors excluded persons with negative or zero household net worth at baseline from the analysis.	Yes. Adverse wealth shock was related to a significant increase in depressive symptoms, controlling for health and sociodemographic factors. Persons who had experienced wealth shock reported significantly higher depressive symptoms than persons who had not experienced a wealth shock.
Celeste and Fritzell (2018)	How do absolute and relative socioeconomic inequities in musculoskeletal pain, oral health and psychological distress evolve with aging?	Adults ages 15–62 at baseline studied over the lifecourse in the Swedish Level‐of‐Living Survey and the Swedish Panel Study of Living Conditions of the Oldest Old. Six waves spanning 43 years from Wave 1 (1968) to Wave 6 (2010–2011) included from five cohorts 1906−1915 (*n* = 1050), 1925−1934 (*n* = 989), 1944–1953 (*n* = 1235), 1957–1966 (*n* = 1042) and 1970–1981 (*n* = 1199). Final analytic sample: *N* = 4487. Sweden.	Observational prospective cohort study on nationally representative panel data.	Past 12‐month psychological distress. Defined by responses to question asking, “In the last 12 months, have you suffered from insomnia, overexertion, mental illness, depressions or deep dejection and/or nervous trouble (anxiety, uneasiness, anguish)? Responding ”yes, severe“ to one or more or ”yes, mild” to three or more of these conditions was coded as severe psychological distress.	Yes. Lack of cash margin defined by response to the following question, “If a situation suddenly arose where you had to come up with SEK, could you manage it?” with a yes/no response. The value of Swedish crowns (Kr) was adjusted over time. In 1968, Kr 2000 was listed, and in 2010, Kr 14000 (US$1950 or €1450) was listed. Person who answered “yes” in all waves of the study were coded as “never poor.” Persons who responded “no” at least once were coded as “at least once poor.”	Yes. Persons who had been poor at least once in their life had higher psychological distress across the lifecourse than persons who had never been poor. Absolute differences in psychological distress were observed across the lifecourse. While relative inequities got smaller over the lifecourse, they still persisted into old age.
Slater et al. (2018)	Are metabolic health or obesity associated with depression among older adults in England? What independent associations exist between depression and covariates over 2‐year follow‐up?	Older adults ages 50 and over in the English Longitudinal Study of Ageing (ELSA) study. Wave 1 (2012–2013); through Wave 2 (2014–2015) used. *N* = 6804. UK.	Observational prospective cohort study on nationally representative panel data.	Past week depressive symptoms. CES‐D eight‐item scale. Elevated depressive symptoms defined as a dichotomous variable for a score of 4 or more.	Yes. Wealth quintiles included household wealth (e.g., financial assets, physical assets, and housing wealth) and excluded pension wealth. Household wealth calculated by summing assets, including assets held in bank accounts in England, the value of owner‐occupied housing (minus outstanding mortgage), the value of business properties or holiday homes (minus outstanding mortgages), and the value of physical assets such as jewelry, artwork, and antiques, and by subtracting debts.	Yes. Persons in wealth quintiles 3,4, and 5 had significantly lower odds of developing elevated depressive symptoms at 2‐year follow‐up than persons in quintile 1 (*p* < .01). Persons in quintile 2 had lower odds of depressive symptoms at follow‐up than persons in quintile 1 but the results were not statistically significant (*p* < .07).
Boyce et al. (2018)	Do central bank interest rate changes directly influence mental health, and does the effect vary by level of indebtedness?	Adults in the British Household Panel Survey (BHPS). Study used 14 waves of data. Wave 1 (1995) through Wave 14 (2009). Final analytic sample: *N* = 15,818. UK.	Observational prospective cohort study on nationally representative panel data.	Recent psychological distress. General Health Questionnaire (GHQ) 12‐item scale. Authors measured mental health in two ways: (1) psychological distress, measured as a continuous variable and (2) psychological case‐ness, as a binary indicator score above 3 on a 0–12 scale.	Yes. Household savings defined as a binary indicator in response to this question, “Do you save any amount of your income for example by putting something away now and then in a bank, building society, or Post Office account other than to meet regular bills?” Household debt position defined by response to two questions: (1) “Do you or anyone in your household have to make repayments on hire purchases or loans?” where participants were asked to include home mortgage loans but to exclude Department of Social Security social fund loans. (2) Participants were asked “to what extent is the repayment of such debts and the interest a financial burden on your household?” where responses included ”heavy burden,” ”somewhat of a burden,” and ”not a problem.” Interest rates defined by the Bank of England base‐rate on the day each person was interviewed. As an alternative interest rate indicator, authors also looked at the average interest rate in the year up until the person's interview.	Complicated. Being a saver was associated with a lower prevalence of psychological distress or psychological case‐ness but the relation was not statistically significant. Interest rates on average were not linked to mental health; however, the influence of interest rates on mental health varied by household debt position. While there appeared to be a negative effect on mental health for persons with heavy debt burden when interest rates were high, the results were not statistically significant.
Bogan and Fertig (2018)	Does psychological distress have an effect on retirement account behavior? Does the relation vary by marital status?	Adults in the Panel Study of Income Dynamics (PSID) and older adults in the Health and Retirement Study (HRS). Sample restricted to men below the age of 65 years and women below the age of 67 years. PSID: Waves from 2001 to 2013. HRS: Waves from 1996 to 2012. PSID *N* = 10,770. HRS *N* = 8201. USA.	Observational prospective cohort study on two sets of nationally representative panel data; using a lagged model (2 years between waves) to see how change in depression effects change in retirement savings.	Past 30‐day psychological distress (PSID). Kessler‐6 scale. Measured moderate psychological distress (score of 5 through 12) and severe psychological distress (score of 13 or higher). Past week depressive symptoms (HRS). Center for Epidemiological Studies’ Depression Scale (CES‐D) eight‐item scale. Moderate mental illness defined by a score of 4 or higher. In secondary analyses, an indicator of previous diagnosis of depression was used.	Yes. Retirement account data captured in multiple ways. Authors measured having any voluntary retirement savings vehicle (looking specifically at defined contribution pension plan (DC), Independent Retirement Account (IRA), and Keogh accounts). Share of financial assets devoted to these accounts and financial distress scale (created by dividing consumer debt by income) were measured.	Yes. Reporting greater psychological distress was associated with a large and significant effect on behavior of saving money; reporting psychological distress was associated with a lower probability of having a retirement account, with lower total retirement savings amounts as a portion of assets, and with more retirement fund withdrawals. Persons who were unmarried reported having a lower share of assets for retirement than married persons, regardless of mental health status.
Tankard et al. (2019)	Can an economic empowerment intervention improve women's social empowerment, IPV victimization, and health?	Female adults ages 18–55 years who were low income and living in urban parts of Columbia who had not used informal or formal savings services or bank services in the past 12 months. Wave 1 (2013) Wave 2 (9 months after baseline) Wave 3 (18 months after baseline). *N* = 1800. Columbia.	Experimental study: randomized control trial with economic empowerment intervention on a group of urban poor women in Columbia.	Symptoms of psychological distress. Self‐report scales (0–4 scale) of depression measured at provider health checkup.	Yes. Bundled treatment for intervention included free health checkups and a free personal savings account at neighborhood local banks. Participants in the treatment group received an initial deposit of 10,000 pesos ($5 US dollars) and matching 1:3 funds up to a limit.	Yes. Women in the savings intervention arm had statistically significant lower rates of depression than women in the control arm. The treatment effects were largest for women whose baseline surveys did not reflect intimate partner violence. From the 1364 women allotted to treatment, 49% created an account, 33% made one deposit or more, and 21% made one withdrawal or more. The median total deposited across the project of $95 US dollars.
Karimli et al. (2019)	How does participation in the bridges to the future intervention influence mental health among AIDS‐orphaned children? How do household wealth, child poverty, and child work mediate the effect of the intervention on child mental health?	Youth ages 10–16 years who had lost one or both parents due to AIDS and who were enrolled in school. Wave 1 (baseline), Wave 2 (12 months after baseline), Wave 3 (24 months after baseline), Wave 4 (36 months after baseline), and Wave 5 (48 months after baseline). *N* = 1410. Uganda.	Experimental study: randomized control trial with microfinance intervention on a group of AIDS‐orphaned children with two treatment arms and a control group.	Past 2‐week depressive symptoms. Children's Depression Inventory (CDI) 24‐item scale. Depression defined as a binary indicator at a cutoff score of 12 or more.	Yes. Child poverty was defined by a cumulative score (ranging from 0 to 6) summing six binary variables indicating whether the child has more than two sets of clothes; a blanket; at least two pairs of shoes; had meat/fish at least once in the last week; had three meals per day in the last 7 days; and had tea with sugar at least once in the last 7 days. The household wealth index was created by standardizing indicator variables, calculating the factor loadings, and multiplying the indicator variables by the loadings and summing the value to produce a household index value. There were two intervention groups. Treatment arm 1 received economic strengthening and asset accumulation assistance with a 1:1 savings match (called Bridges). Treatment arm 2 received economic strengthening and asset accumulation assistance with a 2:1 savings match (called Bridges PLUS).	Complicated. The treatment group reported lower rates of child poverty at 12 months and a significantly lower depression after 24 months. At 36 months and 48 months, the treatment group showed lower child depression, but it was not statistically significant. The treatment effect did not vary based on household wealth.
Kilburn et al. (2019)	Does economic well‐being effect sexual behavior and well‐being? Do economic intervention program effects vary by household socioeconomic status?	Young females ages 13–20 attending high school participating in a Conditional Cash Transfer program for education. Wave 1 (2012). Wave 2 (12 months after baseline), Wave 3 (24 months after baseline), Wave 4 (36 months after baseline). *N* = 2537. Ghana.	Experimental study: randomized control trial with conditional cash transfer intervention on school attending female adolescents with one treatment arm and a control group.	Past week depressive symptoms. Center for Epidemiological Studies’ Depression Scale (CES‐D) 20‐item scale.	Unclear. The study notes that young women “had savings.” Financial support provided to treatment arm in the form of a monthly cash transfer to girls and their parents (with 2:1 contribution to parental accounts). Cash transfers for girls and their parents provided if girls attended 80% or more of school days in the past month. Monthly contributions equaled 100 Rand for young women ($10 US Dollars) and 200 Rand for parents ($20 US Dollars).	Complicated. The conditional cash transfer program (CCT) had significant effects on improving depressive symptoms among women in below median household income groups but not among women in above median household income groups. The cash transfer had larger effects on psychological well‐being among participants with below median level household income at baseline.

#### 3.2.4 Observational studies following macro‐economic events

Seven studies used observational designs following macro‐economic events such as shifts in the stock market, housing prices, or interest rates. The majority of articles using macro‐economic events as focal points of their design used older adult study populations (*n* = 5) (ages 50 years and old); only two articles (*n* = 2) using macro‐economic as focal points of their design had study populations ages 18 years and older. Among studies following macro‐economic events, three reported an inverse relation between wealth and depression (Hamoudi & Dowd, 2014; Pool et al., 2017; Yilmazer et al., 2015). Pool et al. (2017) reported that adverse wealth shock was associated with an increase in depressive symptoms up to 4 years after the wealth shock. Yilmazer et al. (2015) reported that losses in housing and nonhousing wealth were associated with an increase in psychological distress. Meanwhile, Hamoudi and Dowd (2014) found that increased housing values were associated with improved psychological health, with greatest effects for home owners.

Four studies focusing on macro‐economic events reported “complicated” findings (Boyce et al., 2018; Cagney et al., 2014; McInerney et al., 2013; Wilkinson, 2016). In general, these studies found that certain subgroups reported an inverse relation between wealth and depression and that other subgroups reported nonsignificant relations. McInerney et al. (2013) found that the 2008 stock market crash had no effect on depressive symptoms of nonstock holders, had a significant negative effect on depressive symptoms among above‐median (wealthier) stock‐holders (leading to more depressive symptoms), and had a *positive effect* on depressive symptoms of below‐median (lower wealth) stock‐holders (leading to less depressive symptoms). Cagney et al. (2014) found that living in a neighborhood with high foreclosure rates was associated with more depressive symptoms among older adults following the 2008 stock market crash and subprime mortgage crisis; however, they found no significant relation between having more assets and depression for people reporting default, auction, or transition to real‐estate. Wilkinson (2016) found that having greater objective indicators of wealth did not appear to predict depressive symptoms following the 2008 Great Recession, but *subjective* indicators such as financial strain did. Boyce et al. (2018) found that low interest rates were inversely related to depressive symptoms among persons with high‐debt but the relation between interest rates and depressive symptoms did not vary significantly by savings status.

#### 3.2.5 Observational studies across the lifecourse

Seven studies explored how wealth influenced depression across the lifecourse. Among these studies, four found an inverse relation between wealth and depression (Bogan & Fertig, 2018; Celeste & Fritzell, 2018; Mossakowski, 2008; Smith et al., 2005). Mossakowski (2008) found that childhood wealth was inversely related with depressive symptoms in early adulthood; in particular, having zero family net wealth or negative family wealth in childhood led to significantly greater depressive symptoms in adulthood. In a study that followed Swedish persons for 43 years, being poor at least once in life led to greater psychological distress across the lifecourse; they also found that while relative differences in psychological distress between poor and never‐poor persons decreased over time, they persisted through old age (Celeste & Fritzell, 2018). Smith et al. (2005) studied the effects of disability onset on depression over time. They found that having wealth protected persons from worse depressive symptoms following onset of disability; these effects appeared to fade after 2 years, with persons having lower wealth recovering mental health following adaption to the disability. Bogan and Fertig (2018) focused specifically on onset of depression and found that persons who developed depression were more likely to have less retirement savings over time than their peers who did not develop depression.

Two studies across the lifecourse showed complicated relations between wealth and depression (Hraba et al., 2001; Lê‐Scherban et al., 2016). Lê‐Scherban et al. (2016) studied the influence of childhood wealth on depressive symptom severity into early adulthood. They found a significant relation for serious psychological symptoms but not for mild and moderate psychological symptoms. For example, young adults in the highest wealth quintile during their childhood had significantly lower odds of serious psychological symptoms than their peers in the lowest childhood wealth quintile. Hraba et al. found that increases in wealth over time were related to reductions in depressive symptoms for women but not for men in the Czech Republic.

Finally, one study assessing aging found no significant results: Silveira et al. (2005) used proxy reports to assess wealth and depression in the last year of life. They found no significant association between depression defined by proxy and wealth, but they did find lower symptom burden of depression among deceased persons with greater wealth.

#### 3.2.6 Observational studies following job loss

Four studies observed the association of wealth and depression following job loss. One study found an inverse relation between wealth and depression: Gallo et al. (2006) reported that among persons with below median nonhousing wealth, depressive symptoms 4 and 6 years after job loss were significantly greater among persons than among persons who had not lost their jobs; among persons with above median nonhousing wealth, there was no significant difference in reporting of depressive symptoms between persons who experienced job loss and persons who did not. Two studies reported complicated findings. Rodriguez et al. (1999) found that wealth was associated with depressive symptoms following job loss for persons who were white but not for African‐American persons. Riumallo‐Herl et al. (2014) assessed depressive symptoms following job loss in the United States and Europe; they found that depressive symptoms scores were higher following job loss across both regions, but the effect was greater in the United States. They found no significant interaction between job loss and wealth in depression in Europe but they did find a significant interaction in the United States; namely, persons with little or no wealth before losing their jobs experienced significantly more depressive symptoms than their counterparts with wealth.

One study found no significant relation between wealth and depression following job loss: Berchick et al. (2012) found no significant relation between wealth and depression after unexpected job loss; they did find that having lower education and higher occupation prestige were associated with greater risk of depression following job loss.

#### 3.2.7 Observational studies measuring general patterns between wealth and depression

Seven studies assessed general trends between wealth and depression to understand how wealth was associated with depression in the normal course of living. Three studies reported an inverse relation (Carter et al., 2009; McGovern & Nazroo, 2015; Slater et al., 2018), two studies reported a complicated relation (Dew, 2007; Hounkpatin et al., 2015), one study found no significant relation (Yoshikawa et al., 2008), and one study found a direct relation (Nieuwenhuis et al., 2017). In New Zealand, Carter et al. (2009) specifically sought to answer the question of whether wealth was associated with mental health (and whether that relation held when controlling for other socioeconomic indicators). They found that having more wealth was significantly associated with having fewer psychological symptoms over time; this association was greater than that between household income and psychological symptoms. McGovern and Nazroo (2015) sought to explore the relation between wealth and mental health to understand potential mechanisms for the relation; in a study of UK older adults, they found a direct effect of wealth on depression. Further, the relation between objective indicators of wealth and depression was partially mediated by subjective indicators such as social status. Slater et al. (2018) focused their research question on metabolic health, obesity, and depression, but they did report relations with covariates and in doing so found that persons in the three highest wealth quintiles were less likely to report depressive symptoms after 2 years than persons in the lowest wealth quintile. Of the studies that found a complicated relation, Dew (2007) found that wealth and depressive symptoms were inversely related in unadjusted models but that the association was no longer significant when controlling for other factors, suggesting that the relation was mediated by other factors. In a study of older adults in the United States and the United Kingdom, Hounkpatin et al. (2015) found that wealth was associated more strongly with depressive symptoms in the United Kingdom than in the United States; they also found that wealth rank was more important than absolute wealth in predicting depressive symptoms over time. In a study assessing access to institutional resources (owning a savings account), Yoshikawa et al. (2008) found no significant relation between having access to a checking account or savings account and depressive symptoms; they did find that institutional resource access was related to reduced financial distress, which in turn was associated with depressive symptoms. In the one longitudinal study that showed a direct relation between wealth and depression, Nieuwenhuis et al. (2017) found that teens who moved to higher wealth neighborhoods had a significantly higher likelihood of reporting depression than teens who had not moved.

#### 3.2.8 Experimental studies

Seven experimental studies reported on savings interventions that aimed to improve mental health. Among the seven experimental studies, five reported an inverse relation between wealth and depression, where participants reported significantly lower depression following the savings interventions (Han et al., 2013; Huang et al., 2014; Karasz et al., 2015; Ssewamala et al., 2012; Tankard et al., 2019). The five studies showing an inverse relation were conducted among adults (*n* = 3) and children (*n* = 2) and were conducted in the United States (*n* = 2), Uganda (*n* = 2), and Columbia (*n* = 1). The five successful interventions had multiple components; in addition to savings incentives (through matching contributions in savings accounts) they included educational or social components through mentorship programs (Han et al., 2013; Ssewamala et al., 2012) or peer coaching (Karasz et al., 2015) to encourage saving. Mothers in the treatment arm of the SEED Oklahoma intervention, which provided initial funds for their young children's college accounts and matching contributions, reported lower depressive symptoms than women in the control arm (Huang et al., 2014). Of the two articles that reported more complicated results, one showed a treatment effect (significantly lower depression in the savings intervention group) at 12‐ and 24‐month of follow‐up that attenuated at 36‐ and 48‐month follow‐up (Karimli et al., 2019). The second article that reported complicated results found that the savings intervention resulted in significantly lower depression at follow‐up for women with below median household income but not for women with above median household income.

In the experimental studies, context mattered for the relation between wealth and depression. Among the seven experimental interventions, household context affected the treatment effect in four of the studies (Han et al., 2013; Huang et al., 2014; Kilburn et al., 2019; Tankard et al., 2019). At times, this could improve or weaken the intervention effect. In the case of a conditional cash transfer in Ghana, the women who benefited most from the cash savings intervention were those who had below median income households (Kilburn et al., 2019). In the SEED Oklahoma study, treatment effects were significantly larger for women who were low‐income and had low education (Huang et al., 2014). In Colombia, the effects of participating in a savings intervention were greatest among women who had not experienced intimate partner violence at baseline (Tankard et al., 2019).

## 4 DISCUSSION

### 4.1 Summaries of the characteristics of the full review and charted review

There is a growing literature on wealth and depression. We identified 96 articles that have been published from the start of online search history through July 19, 2020 that featured the relation between wealth and depression. The number of publications on wealth and mental health has increased over time, and the number of longitudinal studies also increased over time.

#### 4.1.1 Comparison of characteristics of longitudinal versus cross‐sectional studies

The characteristics of longitudinal studies and cross‐sectional studies were similar. Both featured majority adult populations, with greater than one third of studies featuring only older adults. One difference between longitudinal articles and cross‐sectional studies was that the proportion of studies conducted outside of the United States was greater among cross‐sectional studies than among longitudinal studies. Although cross‐sectional studies do not allow for causal inference, they nevertheless provide important information about the link between wealth and depression. And, in particular, they can help us to understand how the relation between wealth and depression may vary across countries until more longitudinal studies are established globally. Both longitudinal and cross‐sectional studies signaled consistent patterns, namely, that greater wealth had a significant association with less depression across a range of contexts, populations, and scenarios.

### 4.2 Summaries of the findings of the full review and charted review

The majority of studies in both the full review and the charted review showed an inverse relation between wealth and depression. In general, having more assets was associated with less depression across the studies reviewed. Across the world, age groups, genders and races, having more assets was related to less depression throughout the lifecourse. More than one half of studies reported an inverse relation between wealth and depression and one third of studies reported complicated relations between wealth and depression. While the associations between wealth and depression were not always statistically significant, the relation between the two constructs, with rare exception, showed a dose response pattern, where more wealth being associated with less depression.

### 4.3 Charted review

The charted review featured 32 longitudinal articles on wealth and depression. Almost half of the articles included (*n* = 15, 47%) used data from nationally representative longitudinal studies conducted in the United States. The majority of articles used datasets from population‐representative panel studies, reflecting the importance of large, nationally funded longitudinal datasets in allowing for comparisons of wealth and depression over time.

Articles in the longitudinal charted review could be grouped into five categories: observational studies following macro‐economic events, observational studies across the lifecourse, observational studies following job loss, observational studies measuring general patterns between wealth and depression, and experimental studies. Within these groupings, additional subthemes emerged, namely, (1) macro‐economic events influenced depression, (2) wealth status influenced depression across the lifecourse, (3) wealth protected against depression in the face of stressors such as job loss, (4) subjective or psychosocial factors such as perception of wealth, relative comparison, and social status modified the relation between wealth and depression, and (5) savings interventions were successful in reducing depression and varied by context.

#### 4.3.1 Observational studies following macro‐economic events

Economic events influence mental health; shifts in the stock market, interest rates, and value of housing influenced mental health for years to follow. These findings were consistent with other reviews that have looked at the effects of economic crises on mental health (Haw et al., 2015; Uutela, 2010; Vásquez‐Vera et al., 2017).

#### 4.3.2 Observational studies across the lifecourse

Economic experiences early in life influenced mental health throughout the lifecourse and inequities persisted through old age. Studies of young adults found that wealth in childhood or adolescence affected health later in life (Lê‐Scherban et al., 2016; Mossakowski, 2008). The studies that included children also reported dynamic relations among household members; for example, the SEED Oklahoma child college savings initiative resulted in improved mental health for mothers who could not use the funds themselves (Huang et al., 2014). Thus, intervening earlier in the lifecourse may yield dividends in the years to follow on mental health for the individuals themselves and their caregivers.

#### 4.3.3 Observational studies following job loss

Existing studies suggest that having wealth protected against depressive symptoms up to 4–6 years following job loss; differences in depressive symptoms following job loss between Europe and the United States may indicate that broader social safety net policies in Europe may have softened the blow of job loss. Given the much less robust economic and social safety net following job loss in the United States, people must rely more on personal savings, which may, therefore, explain the significant interactive effects of wealth and job loss on depression in the United States. These findings are consistent with a cross‐sectional study that suggests that the reduced influence of personal wealth on depression in Nordic countries may be due to generous social and economic safety nets in those countries (Kourouklis et al., 2019). Future policies, therefore, may wish to focus on savings interventions to reduce the mental health effects of job loss.

#### 4.3.4 Observational studies measuring general patterns between wealth and depression

These studies suggested that subjective or psychosocial factors may be driving part of the relation between wealth and depressive symptoms. The studies together suggest that rank (Hounkpatin et al., 2015), social status (McGovern & Nazroo, 2015), and relative wealth compared to others in one's neighborhood (Nieuwenhuis et al., 2017) all influence the relation between wealth and depression.

#### 4.3.5 Experimental studies

Savings interventions made a significant difference, particularly among specific subpopulations. The experimental studies showed that interventions to increase wealth can work, and that their effectiveness is in part determined by context. Treatment effects varied by level of social or economic well‐being prior to intervention. As with all other determinants of health, wealth is one of many structural and significant factors that shapes health outcomes. While these studies showed that wealth was a predictor of depression, and one that could be intervened upon, they also highlighted the complex set of causal factors that shape the contexts people live in and that shape health. Wealth interventions should balance the full suite of factors that contribute to health, and the trade‐offs inherent in investing in particular interventions over others.

### 4.4 Other reviews on wealth and health

This review is consistent with other reviews assessing the relation between wealth and mental health. The most relevant example is a systematic review of studies on wealth and health from 1990 through 2006 conducted by Pollack and colleagues. Of the six studies they included that examined the relation between wealth and mental health, five reported significant links between wealth and mental health within at least a subgroup of the population. One study found no significant association between wealth and depression once controlling for other factors. They concluded that wealth was a significant factor in health, and recommended that wealth and income be measured separately (Pollack et al., 2007).

Our findings that assets across the life course were associated with reduced depression were consistent with reviews on other socioeconomic indicators and mental health outcomes. Muntaner (2004) reviewed the literature on mental disorders and socioeconomic position. The author found in general that having a higher socioeconomic position was associated with better mental health; in particular, they found that persons in lower social strata consistently had higher depression. Similar to our findings, they identified exposure across the lifecourse as a central and important question to consider when understanding the influence of socioeconomic status on depression. Mair et al. (2008) conducted a review of neighborhood characteristics and depressive symptoms; variability in studies made it difficult for them to make conclusions about definitive relations between socioeconomic neighborhood qualities and depressive symptoms. Osypuk et al. (2014) conducted a review of economic and social policies and their influence on health broadly, and found evidence of long‐term effects of various public policies on mental health. Thus, our findings that wealth across the lifecourse affects depression is consistent with findings by others.

### 4.5 Limitations

This scoping review has several limitations. First, we limited our reviews to articles that included an objective wealth indicator (e.g., financial assets or savings). We did not include articles that only had subjective indicators of wealth (e.g., financial strain) although subjective indicators may well explain some of the relation between assets and mental health. Second, we also did not include studies that featured debt without other objective indicators of wealth, given that debt as a concept has a complex relation with depression (Richardson et al., 2013), meriting a separate investigation. However, understanding objective indicators is an important part of the complex set of factors that contribute to depression, so these findings can help pave the way for future work on other aspects of financial assets. Third, our results reflect the literature as of July 2020 which may change and evolve over time. Finally, the studies included were limited to English language studies, which may exclude findings conducted globally.

### 4.6 Recommendations for future research

We highlight three main areas for future research based on our findings. First, more longitudinal panels should collect data on mental health and wealth over time. This may entail either creating new longitudinal studies, particularly outside of the United States, or simply consistently adding questions about wealth and depression to existing studies. Including wealth as a feature in data collection can provide a rich and important set of information about a person's ability to withstand stressors. Studies about wealth often center on a change in wealth status over time, either caused by an exogenous force such as a macro‐economic event or an intervention such as a savings inventive program. With that in mind, measuring depression and wealth before events occur is critical for having a baseline mental health and understanding how changes in wealth affect depression over time. Having established longitudinal cohorts that measure wealth and depression would allow researchers to understand these relations at individual points in time, to be able to capture changes in mental health following changes in depression (or vice versa), and would allow for effective policy interventions to reduce the ill effects of negative wealth changes on depression.

Second, studies on wealth should define how the concept is operationalized, and strive towards consistency with established work in order to build a more robust body of evidence. Studies should include contextual factors, potentially measuring objective and subjective indicators of wealth, to better understand the mechanism and causal drivers of mental health. This review showed that more than one third of articles concluded that the relation between wealth and depression is complicated, suggesting that having more information on the definition and types of assets may provide clarity on the role of assets in shaping mental health.

Third, more intervention studies should be conducted to assess how savings initiatives can improve mental health in different contexts across different populations. Interventions can reduce depression, and studies show several examples that have been met with success. There is much work to be done in the future to explore the relative benefits of particular interventions, weighing considerations such as return on investment and equity versus efficiency trade‐offs.

## 5 CONCLUSIONS

The preponderance of evidence reviewed here makes the case that wealth provides an important factor to consider when understanding the full economic and social context shaping the mental health of populations. Above and beyond income, assets may provide a clearer picture of a person's financial stability and psychological protective or risk factors. The literature has evolved over time, with an increase in articles including wealth in the past decade. Expanding the literature on mental health and wealth holds promise. Having more longitudinal data about mental health and wealth would help improve our understanding of the causal drivers of mental health and could allow us to address growing health inequities. The COVID‐19 pandemic and its economic effects have yielded the most unequal recession in history, with low‐income persons ending the pandemic with record job loss and financial insecurity. Rising inequalities globally make the call for this work even more urgent, for the research and policy community to better understand the influence of assets towards the end of mitigating growing mental health gaps.

### PEER REVIEW

The peer review history for this article is available at https://publons.com/publon/10.1002/brb3.2486

## Supporting information



Supporting Information

Supporting Information

Supporting Information

## Data Availability

Data sharing is not applicable to this article as no new data were created or analyzed in this study.
