# Supplementary material for: Wealth and depression: A scoping review
Source: Brain Behav. 2022 Feb 8;12(3):e2486. doi: 10.1002/brb3.2486 (PMC8933775; doi:10.1002/brb3.2486)
Supplement: Supplementary file 1 — Supporting Information [file BRB3-12-e2486-s002.pdf]

Supplemental Table 1. Data sources used for longitudinal studies in charted review (n=32)

Data source	Frequency (N)	Percent (%)
Other	9	28%
Health and Retirement Study (HRS)	8	25%
English Longitudinal Study of Ageing (ELSA)	2	6%
National Survey of Families and Households (NSFH)	2	6%
Panel Study of Income Dynamics (PSID)	2	6%
Health and Retirement Study (HRS) and Panel Study of Income Dynamics (PSID)	1	3%
English Longitudinal Study of Ageing (ELSA) and Wisconsin Longitudinal Study (WLS)	1	3%
Swedish Panel Study of Living Conditions of the Oldest Old (SWEOLD) and Level of Living Survey (LNU)	1	3%
National Longitudinal Surveys (NLS)	1	3%
Survey of Families, Income and Employment (SoFIE)	1	3%
Americans' Changing Lives (ALC)	1	3%
Conflict and Management of Relationships (Conamore)	1	3%
British Household Panel Survey (BHPS)	1	3%
National Social Life, Health, and Aging Project (NSHAP)	1	3%

Other: Data collected specifically for the study (including the seven experimental studies and two observational studies).
