# Supplementary material for: Wealth and depression: A scoping review
Source: Brain Behav. 2022 Feb 8;12(3):e2486. doi: 10.1002/brb3.2486 (PMC8933775; doi:10.1002/brb3.2486)
Supplement: Supplementary file 2 — Supporting Information [file BRB3-12-e2486-s001.pdf]

Appendix A. Search Strategy

Searches conducted July 19, 2020

Pubmed search:

("Depressive Disorder"[Mesh] OR Depression[tw] OR "Psychological Distress"[Mesh] OR psychological distress[tw] OR depress*[tw] OR Common mood disorder[tw]) AND ("Economic Status"[Mesh] OR wealth[tw] OR savings[tw] OR financial strain[tw] OR debt[tw])

Filters applied: humans; English language

Ebsco search, in Medline, PsycINFO, PsycArticles, SocINDEX, and EconLit:

(Depression OR depressive OR psychological distress OR depress* OR Common mood disorder) AND (wealth OR financial strain OR savings OR debt)

Select: Scholarly (Peer Reviewed) Journals; English language; human subject

Embase search:

#1 'depression'/exp OR depression
#2 depression OR depressive OR 'psychological distress' OR depress* OR 'common mood disorder' OR 'psychiatric diagnosis'
#3 #1 OR #2
#4 wealth OR 'financial strain' OR savings OR debt
#5 #3 AND #4
#6 #3 AND #4 AND ([article]/lim OR [article in press]/lim) AND [english]/lim

Combined
