# Supplementary material for: Wealth and depression: A scoping review
Source: Brain Behav. 2022 Feb 8;12(3):e2486. doi: 10.1002/brb3.2486 (PMC8933775; doi:10.1002/brb3.2486)
Supplement: Supplementary file 3 — Supporting Information [file BRB3-12-e2486-s003.pdf]

Appendix B. Full Review Articles (n=96)

Articles included in the full review: authors, years, and study design. Longitudinal studies featured in the charted review.

Author	Year	Study design
Abas MA, Punpuing S, Jirapramukpitak T, Guest P, Tangchonlatip K, Leese M, Prince M	2009	Cross-sectional
Anand P, Esposito L, Villaseñor A	2018	Cross-sectional
Arrindell WA, Steptoe A, Wardle J	2003	Cross-sectional
Back JH, Lee Y	2011	Cross-sectional
Berchick ER, Gallo WT, Maralani V, Kasl SV	2012	Longitudinal
Bhan N, Millett C, Subramanian SV, Dias A, Alam D, Williams J, Dhillon PK	2017	Cross-sectional
Bogan VL, Fertig AR	2018	Longitudinal
Boyce CJ, Delaney L, Ferguson E, Wood AM	2018	Longitudinal
Cagney KA, Browning CR, Iveniuk J, English N	2014	Longitudinal
Carter KN, Blakely T, Collings S, Gunasekara FI, Richardson K	2009	Longitudinal
Cazzuffi C, López-Moreno D	2018	Cross-sectional
Celeste RK, Fritzell J	2018	Longitudinal
Chen D, Petrie D, Tang K, Wu D	2018	Cross-sectional
Christensen S, Zachariae R, Jensen AB, Vaeth M, Moller S, Ravnsbaek J, von der Maase H	2008	Cross-sectional
Decaro JA, Manyama M, Wilson W	2016	Cross-sectional
Demakakos P, Nazroo J, Breeze E, Marmot M	2008	Cross-sectional
Dew J	2007	Longitudinal
Docrat S, Cleary S, Chisholm D, Lund C	2019	Cross-sectional
Ettman CK, Cohen GH, Galea S	2020	Cross-sectional
Evrou M, Falkingham JC, Qin M, Vlachantoni A	2017	Cross-sectional
Familiar I, Murray S, Ruisenor-Escudero H, Sikorskii A, Nakasujja N, Boivin MJ, Opoka R, Bass JK	2016	Cross-sectional
Flynn EP, Chung EO, Ozer EJ, Fernald LCH	2017	Cross-sectional
Gallo WT, Bradley EH, Dubin JA, Jones RN, Falba TA, Teng, HM, Kasl SV	2006	Longitudinal
Ganzini L, McFarl BH, Cutler D	1990	Cross-sectional
Geldsetzer P, Vaikath M, Wagner R, Rohr JK, Montana L, Gomez-Olive FX, Rosenberg MS, Manne-Goehler J, Mateen FJ, Payne CF, Kahn K, Tollman SM, Salomon JA, Gaziano TA, Barnighausen T, Berkman LF	2018	Cross-sectional
Hailemichael Y, Hanlon C, Tirfessa K, Docrat S, Alem A, Medhin G, Fekadu A, Lund C, Chisholm D, Hailemariam D	2019	Cross-sectional
Halsted S, Ásbjörnsdóttir KH, Wagenaar BH, Cumbe V, Augusto O, Gimbel S, Manaca N, Manuel JL, Sherr K	2019	Cross-sectional

Hamoudi A, Dowd JB	2014	Longitudinal
Han CK, Ssewamala FM, Wang JSH	2013	Longitudinal
Henderson C, Diez Roux AV, Jacobs DR Jr, Kiefe CI, West D, Williams DR	2005	Cross-sectional
Himanshu H, Arokiasamy P, Talukdar B	2019	Cross-sectional
Hosseinpoor AR, Bergen N, Mendis S, Harper S, Verdes E, Kunst A, Chatterji S	2012	Cross-sectional
Hounkpatin HO, Wood AM, Brown GDA, Dunn G	2015	Longitudinal
Hraba J, Lorenz FO, Ma E, Pechaçová Z	2001	Longitudinal
Huang J, Sherraden M, Purnell JQ	2014	Longitudinal
Kagotho N Ssewamala FM	2012	Cross-sectional
Kagotho N, Patak-Pietrafesa M, Ssewamala FM, Kirkbride G	2017	Cross-sectional
Kahn JR, Fazio EM	2005	Cross-sectional
Karasz A, Raghavan S, Patel V, Zaman M, Akhter L, Kabita M	2015	Longitudinal
Karimli L, Ssewamala FM, Neils TB, Wells CR, Bermudez LG	2019	Longitudinal
Karmaliani R, Asad N, Bann CM, Moss N, McClure EM, Pasha O, Wright LL, Goldenberg RL	2009	Cross-sectional
Kilburn K, Hughes JP, MacPhail C, Wagner RG, Gómez-Olivé FX, Kahn K, Pettifor	2019	Longitudinal
Kohrt BA, Jordans MJD, Tol WA, Perera E, Karki R, Koirala S, Upadhaya N	2010	Cross-sectional
Kourouklis D, Verropoulou G, Tsimbos C	2019	Cross-sectional
Kulkarni RS, Shinde RL	2015	Cross-sectional
Lachaud J, Hruschka DJ, Kaiser BN, Brewis A	2019	Cross-sectional
Lê-Scherban F, Brenner AB, Schoeni RF	2016	Longitudinal
Lee S, Guo W, Tsang A, Mak ADP, Wu J, Ng KL, Kwok K	2010	Cross-sectional
Lotfaliany M, Hoare E, Jacka FN, Kowal P, Berk M, Mohebbi M	2019	Cross-sectional
Lotfaliany M, Bowe SJ, Kowal P, Orellana L, Berk M, Mohebbi M	2018	Cross-sectional
Lund TJ, Dearing E	2012	Cross-sectional
Marshall A, Jivraj S, Nazroo J, Tampubolon G, Vanhoutte B	2014	Cross-sectional
Martikainen P, Adda J, Ferrie JE, Davey Smith G, Marmot M	2003	Cross-sectional
Maselko J, Bates L, Bhalotra S, Gallis JA, O'Donnell K, Siker S, Turner EL	2018	Cross-sectional
McGovern P, Nazroo JY	2015	Longitudinal
McInerney M, Mellor JM, Nicholas LH	2013	Longitudinal
Montgomery SM, Netuveli G, Hildon Z, Blane D	2007	Cross-sectional
Mossakowski KN	2008	Longitudinal
Muntaner C, Eaton WW, Diala C, Kessler RC, Sorlie PD	1998	Cross-sectional
Nieuwenhuis J, van Ham M, Yu R, Branje S, Meeus W, Hooimeijer P	2017	Longitudinal
Ostrove JM, Feldman P	1999	Cross-sectional
Pool LR, Needham BL, Burgard SA, Elliott MR, de Leon CFM	2017	Longitudinal
Ramlagan S, Peltzer K, Phaswana-Mafuya N	2013	Cross-sectional
Reyes ML, Yujuico IC	2014	Cross-sectional

Richardson LK, Amstadter AB, Kilpatrick DG, Gaboury, MT, Tran TL, Trung LT, Tam NT, Tuan T, Buoi LT, Ha TT, Thach TD, Acierno R	2010	Cross-sectional
Ricketts CF, Rezek JP, Campbell RC	2013	Cross-sectional
Riumallo-Herl C, Basu S, Stuckler D, Courtin E, Avendano M	2014	Longitudinal
Rodriguez E, Allen JA, Frongillo EA Jr, Chra P	1999	Longitudinal
Rodríguez Le Sage M, Townsend A	2004	Cross-sectional
Rwakarema M, Premji SS, Nyanza EC, Riziki P, Palacios-Derflingher L	2015	Cross-sectional
Santric-Milicevic M, Jankovic J, Trajkovic G, Terzic-Supic Z, Babi U, Petrovic M	2016	Cross-sectional
Scholten S, Velten J, Neher T, Margraf J	2017	Cross-sectional
Silveira MJ, Kabeto MU, Langa KM	2005	Longitudinal
Sipsma H, Ofori-Atta A, Canavan M, Osei-Akoto I, Udry C, Bradley EH	2013	Cross-sectional
Slater N, Rowley C, Venables RH, White S, Frisher M	2018	Longitudinal
Smith DM, Langa KM, Kabeto MU, Ubel PA	2005	Longitudinal
Smith ML, Kakuhikire B, Baguma C, Rasmussen JD, Perkins JM, Cooper-Vince C, Venkataramani AS, Ashaba S, Bangsberg DR, Tsai, AC	2019	Cross-sectional
Ssewamala FM, Neils TB, Waldfogel J, Ismayilova L	2012	Longitudinal
Steel N, Hardcastle AC, Bachmann MO, Richards SH, Mounce LTA, Clark A, Lang I, Melzer D, Campbell J	2014	Cross-sectional
Steptoe A, Zaninotto P	2020	Cross-sectional
Stocker R, Nguyen T, Tran T, Tran H, Hanieh S, Biggs BA, Fisher J	2020	Cross-sectional
Tankard ME, Paluck EL, Prentice DA	2019	Longitudinal
Torres JL, Lima-Costa MF, Marmot M, de Oliveira C	2016	Cross-sectional
Tsimbos C	2010	Cross-sectional
Urošević J, Odović G, Rapaić D, Davidović M, Trgovčević S, Milovanović V	2015	Cross-sectional
Valladares-Garrido MJ, Soriano-Moreno AN, Rodrigo-Gallardo PK, Moncada-Mapelli E, Pacheco-Mendoza J, Toro-Huamanchumo CJ	2020	Cross-sectional
Vellakkal S, Millett C, Basu S, Khan Z, Aitsi-Selmi A, Stuckler D, Ebrahim S	2015	Cross-sectional
von dem Knesebeck O, Luschen G, Cockerham WC, Siegrist J	2003	Cross-sectional
Weida EB, Phojanakong P, Patel F, Chilton M	2020	Cross-sectional
Weobong B, Soremekun S, Asbroek AH, Amenga-Etego S, Danso S, Owusu-Agyei S, Prince M, Kirkwood BR	2014	Cross-sectional
Wight RG, Ko, MJ, Aneshensel CS	2011	Cross-sectional
Wikman A, Wardle J, Steptoe A	2011	Cross-sectional
Wilkinson LR	2016	Longitudinal
Xu Y	2011	Cross-sectional
Yilmazer T, Babiarz P, Liu F	2015	Longitudinal
Yoshikawa H, Godfrey EB, Rivera AC	2008	Longitudinal
